# Hypothesis: HPV E6 and COVID spike proteins cooperate in targeting tumor suppression by p53

**DOI:** 10.18632/oncotarget.28823

**Published:** 2026-01-03

**Authors:** Wafik S. El-Deiry

**Affiliations:** ^1^Laboratory of Translational Oncology and Experimental Cancer Therapeutics, Warren Alpert Medical School, Brown University, Providence, RI 02912, USA; ^2^Department of Pathology and Laboratory Medicine, Warren Alpert Medical School, Brown University, Providence, RI 02912, USA; ^3^Joint Program in Cancer Biology, Lifespan Health System and Brown University, Providence, RI 02912, USA; ^4^Legorreta Cancer Center at Brown University, Providence, RI 02912, USA; ^5^Hematology/Oncology Division, Department of Medicine, Lifespan Health System and Brown University, Providence, RI 02912, USA

**Keywords:** HPV, COVID, p53, spike, cancer

## Abstract

Human Papilloma Virus (HPV) is a causative agent in several cancers including cervical cancer, head and neck cancer, anal cancer, penile, vulvar and vaginal cancers. HPV through its virus-encoded protein E6 and the cellular E6-Associated Protein (E6-AP) target the tumor suppressor p53 protein for degradation thereby contributing to cancer development after HPV infection. As viruses cause cancer, the author previously hypothesized that SARS-COV-2 virus may be associated with cancer. More recent insights on the present hypothesis have come from studies suggesting (1) Spike protein of SARS-COV-2 may suppress p53 function, (2) cancer has been associated with mRNA vaccines that produce Spike, and (3) a case mentioned by Dr. Patrick Soon Shiong of a patient who survived HPV-associated head and neck cancer, but the tumor recurred after COVID mRNA vaccination including with liver metastases. Thus, the present hypothesis is that virally encoded proteins such as HPV-E6 or SARS-COV-2 Spike may cooperate in suppressing host defenses including tumor suppressor mechanisms involving p53. The hypothesis can be further explored through epidemiologic and laboratory studies.

It is known that HPV E6 targets the tumor suppressor protein p53 for degradation through the E6-AP thereby contributing to the development of cervical cancer, head and neck cancer, anal cancer and others [1–17].

When the COVID-19 pandemic started, I pursued studies “to better understand and modulate the host immune response to SARSCoV-2 to prevent or reduce disease severity in the current COVID-19 pandemic. Some effort (was) directed at blocking ACE2, the receptor SARS-CoV-2 uses to enter cells.” I further explained by March 24, 2020 (Figure 1) “while the host inflammatory response makes patients critically ill, the host innate immune system including natural killer (NK) cells is involved in fighting and eliminating virally infected cells. Over the last 25 years we have studied this innate immune system pathway that the immune system uses to eliminate transformed and cancer cells as well as virally infected cells. Natural killer cells secrete TRAIL which is involved in killing virally infected as well as transformed cells. This system can be triggered by p53 to suppress viral infection as well as cancer. Thus, our goal is to better understand and modulate the host immune response to increase the innate immune system early in SARS-CoV-2 infection while reducing the severe inflammation that occurs late. We further want to understand the impact of current therapeutics used to treat COVID-19 on both the innate immune system as well as the cellular inflammatory response.”

**Figure 1:**
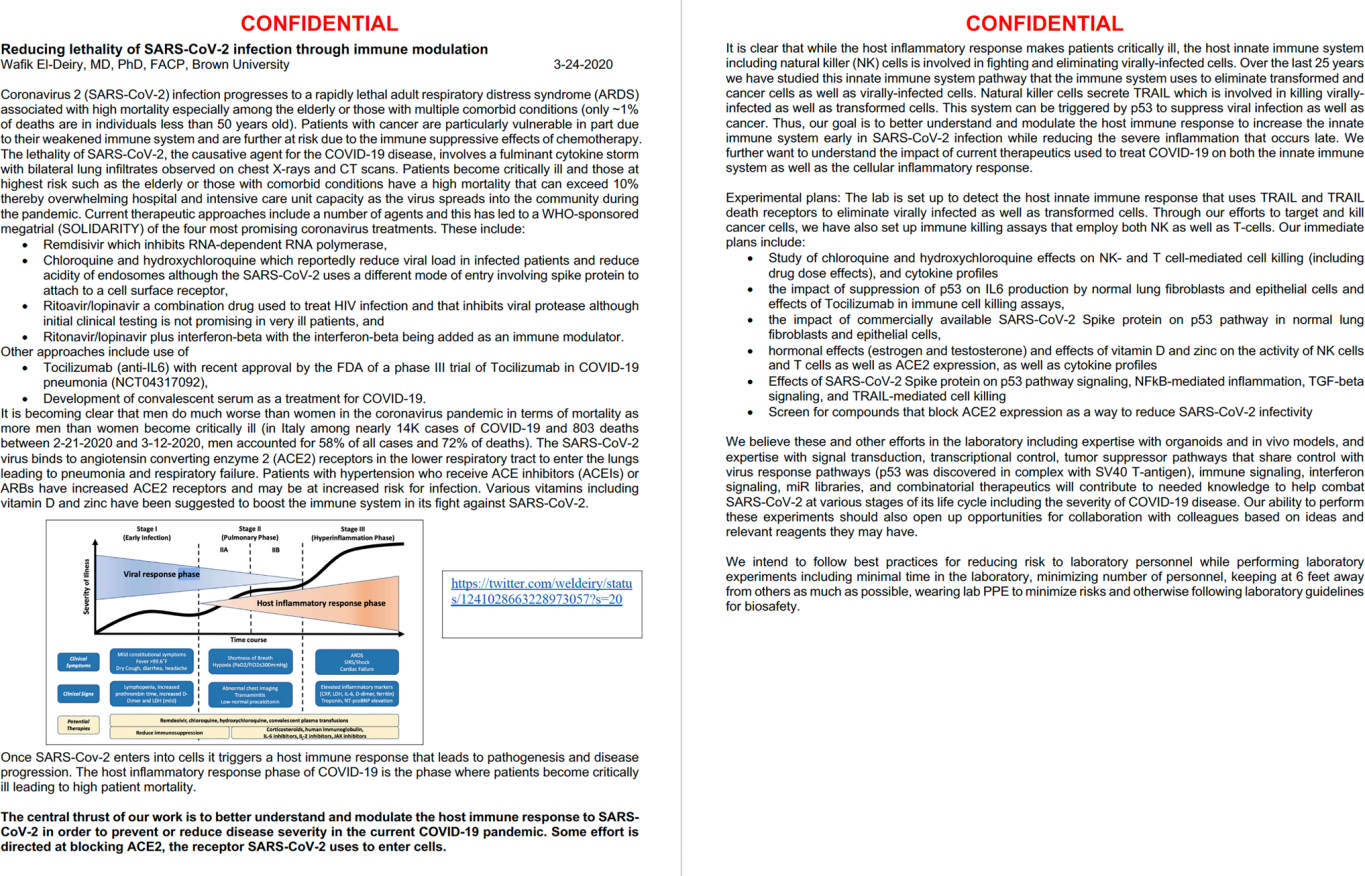
Original seed grant proposal dated 3-24-2020.

The proposal received a Brown University COVID-19 Research Seed Award in the amount of $40,000 for “Reducing the lethality of SARS-CoV-2 infection through immune modulation and drug discovery” in the Spring of 2020. Four publications emerged subsequently from these efforts [18–21]:

2020: MEK inhibitors reduce cellular expression of ACE2, pERK, pRb while stimulating NK-mediated cytotoxicity and attenuating inflammatory cytokines relevant to SARS-CoV-2 infection.
2021: Cytokine ranking via mutual information algorithm correlates cytokine profiles with presenting disease severity in patients infected with SARS-CoV-2.
2022: Integrin/TGF-β1 Inhibitor GLPG-0187 Blocks SARS-CoV-2 Delta and Omicron Pseudovirus Infection of Airway Epithelial Cells In Vitro, Which Could Attenuate Disease Severity.
2024: Transfected SARS-CoV-2 spike DNA for mammalian cell expression inhibits p53 activation of p21(WAF1), TRAIL Death Receptor DR5 and MDM2 proteins in cancer cells and increases cancer cell viability after chemotherapy exposure.

I listened to an interview (https://www.youtube.com/watch?v=tnVMjp9mCA0&t=2s) of Dr. Patrick Soon-Shiong by Chris Cuomo where I learned about a patient named Jim Johnson with a history of HPV-related head and neck cancer who by 2022 had survived his HPV-related cancer for 7 years and then he took the COVID vaccine. The “cancer was back with a vengeance,” and the tumor had metastasized to his liver. After I listened to what happened in this case, it occurred to me that there may be cooperation between HPV and COVID infection or COVID vaccination and suppression of p53.

A search of the literature for “cooperation between HPV and COVID in suppressing p53” found per an AI overview that there is “no evidence of a direct molecular “cooperation” between HPV and COVID-19 in suppressing p53, research indicates they both target the p53 pathway independently, and a COVID-19 infection may indirectly accelerate HPV-related cancer progression by impacting the host immune system.” I found a publication about “SARS-CoV-2 infection heighten[ing] the risk of developing HPV-related carcinoma *in situ* and cancer [22],” and a hypothesis that “COVID-19 can lead to rapid progression of cervical intraepithelial neoplasia by dysregulating the immune system [23].”

## HYPOTHESIS

**Figure 2:**
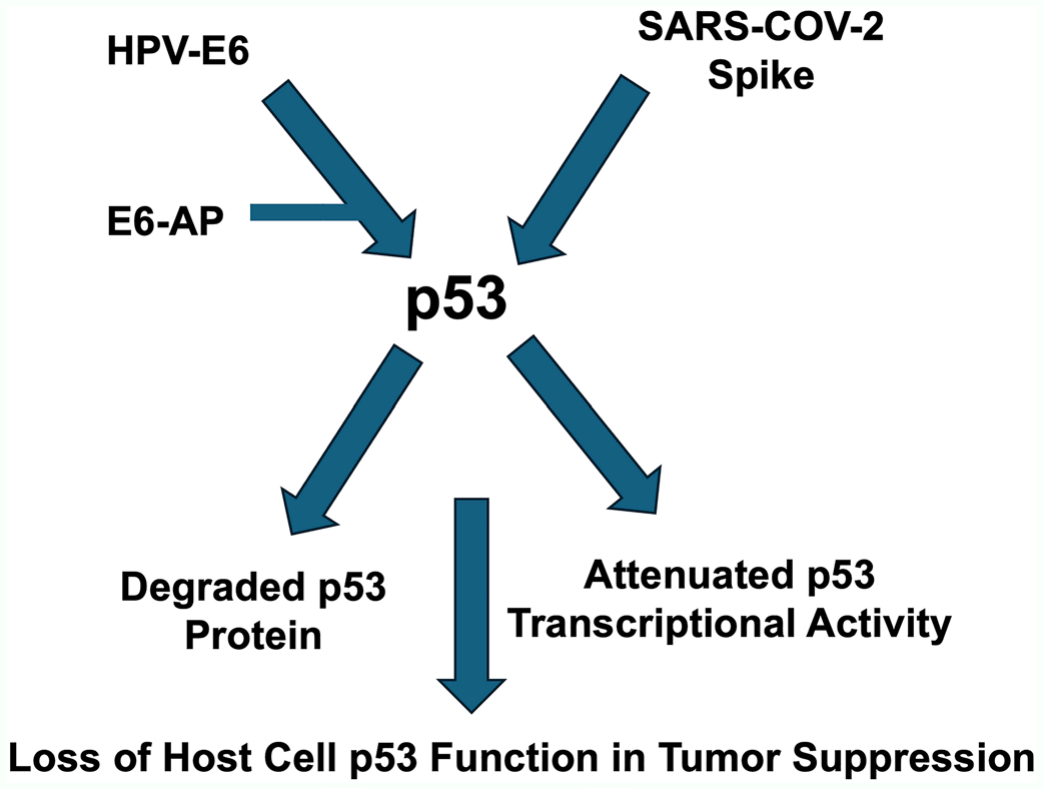
Schematic depicting hypothesized cooperation between HPV and COVID in suppressing p53 and contributing to cancer.

Based on existing literature discussed above, here is a schematic of the hypothesis that HPV E6 and COVID spike proteins may potentially cooperate in targeting tumor suppression by p53 (Figure 2). As depicted in Figure 2, the hypothesis put forth is that virally encoded proteins such as HPV-E6 or SARS-COV-2 Spike may cooperate in suppressing host defenses including tumor suppressor mechanisms involving p53. This hypothesis can be tested through epidemiologic studies looking at cancer incidence and recurrence among HPV-positive individuals who have either been infected by SARS-COV-2 or have been given COVID mRNA vaccines. Laboratory studies can test the impact of HPV-E6 combined with Spike protein on p53 expression and function.
